# Patient-Reported Outcome Measures (PROMs) in Metal-Ceramic and All-Ceramic Fixed Partial Dentures: A Prospective Clinical Study

**DOI:** 10.7759/cureus.103033

**Published:** 2026-02-05

**Authors:** Md Miftah ur Rahman, Bharani Kumar Bhattu, Rahul V.C. Tiwari, S.Y. Rajan, Kapil Laddha, Arjun Sood, Heena Dixit, Seema Gupta

**Affiliations:** 1 Department of Prosthodontics, Crown and Bridge, S.B. Patil Institute for Dental Sciences and Research, Bidar, IND; 2 Department of Dentistry, La Clinica De Familia, Las Cruces, USA; 3 Department of Oral and Maxillofacial Surgery, RKDF Dental College and Research Centre, Bhopal, IND; 4 Department of Oral Medicine and Radiology, Vananchal Dental College and Hospital, Garhwa, IND; 5 Department of Dentistry, Monarch Dental Clinics, Euless, USA; 6 Department of Dentistry, B.R.S. Dental College and Hospital, Pandit Bhagwat Dayal Sharma University of Health Sciences, Rohtak, IND; 7 Department of Blood Cell, Commissionerate of Health and Family Welfare, Hyderabad, IND; 8 Department of Orthodontics, Kothiwal Dental College and Research Centre, Moradabad, IND

**Keywords:** ceramic, fixed partial dentures, metal ceramic, patient-reported outcome measures, quality of life

## Abstract

Introduction

The evaluation of fixed partial dentures (FPDs) has traditionally focused on clinician-centered outcomes, such as survival and technical performance. However, patient-reported outcome measures (PROMs) provide valuable insights into patients’ perceptions of comfort, function, esthetics, and overall oral health-related quality of life (QoL). This study aimed to evaluate and compare patient-reported outcomes in individuals rehabilitated with metal-ceramic and all-ceramic FPDs over a one-year follow-up period.

Materials and methods

This prospective observational clinical study included adult patients who required posterior FPDs. Participants received either metal-ceramic or all-ceramic restorations based on routine clinical decision-making. PROMs were assessed using the Oral Health Impact Profile-14 (OHIP-14) questionnaire and visual analog scales (VAS) for satisfaction assessment. Evaluations were performed at baseline, 6, and 12 months. Clinical parameters, such as chipping, fractures, and secondary caries, were also recorded. Data were analyzed using appropriate non-parametric statistical tests, with a significance level set at p < 0.05.

Results

Both restorative groups demonstrated significant improvements in oral health-related QoL and patient satisfaction following treatment. Baseline PROMs score were comparable between the groups. At the 6- and 12-month follow-ups, all-ceramic restorations showed significantly better patient-reported outcomes than metal-ceramic restorations. Intragroup analysis revealed sustained improvement over time in the all-ceramic group, whereas a partial decline in quality of life scores was observed in the metal-ceramic group at later follow-ups. Chipping was more frequently observed in the all-ceramic group, whereas the fracture rates were comparable.

Conclusion

Both metal-ceramic and all-ceramic FPDs effectively enhanced PROMs; however, all-ceramic restorations provided superior and more stable long-term patient satisfaction and quality of life. Incorporating PROMs into routine clinical evaluations can support patient-centered treatment planning and material selection.

## Introduction

The introduction of patient-reported outcome measures (PROMs) in prosthodontics has shifted the evaluation of dental restorations from purely clinician-centered metrics, such as survival rates and technical complications, to a more holistic assessment that incorporates patients' subjective experiences, including satisfaction, comfort, function, esthetics, and overall oral health-related quality of life (QoL) [1]. In the context of fixed partial dentures (FPDs), which serve as a reliable treatment modality for replacing missing teeth, traditional metal-ceramic prostheses have long been regarded as the gold standard because of their proven long-term clinical performance, with 5-year survival rates often exceeding 94% [2]. These restorations provide excellent mechanical strength and durability, particularly in the posterior regions, but may be limited by esthetic concerns related to metal visibility, potential gingival discoloration, and allergic reactions in sensitive patients.

Advancements in ceramic materials, particularly zirconia-based and other all-ceramic systems, have addressed these limitations by offering superior esthetics, biocompatibility, and a metal-free alternative that aligns with the growing patient demand for natural-looking restorations [3]. All-ceramic FPDs demonstrate promising survival rates, although systematic reviews indicate slightly lower overall survival compared to metal-ceramic counterparts, with higher incidences of technical complications, such as veneer chipping or framework fractures, in certain systems [2,4]. Despite these differences in objective clinical outcomes, patient-centered evaluations often reveal high levels of satisfaction with both material types, particularly regarding esthetics and function, with no significant differences reported [5].

Patient-reported outcome measures provide critical insights into how patients perceive their restorations beyond mere longevity [1]. Studies have shown that patients report high satisfaction with the esthetics and functionality of both metal-ceramic and all-ceramic FPDs, with improvements in QoL after treatment [5,6]. However, comparative data specifically focusing on PROMs between metal-ceramic and all-ceramic FPDs remain limited, highlighting the need for prospective clinical investigations to better understand patient experiences in real-world settings. This prospective clinical study aimed to evaluate and compare the PROMs of patients treated with metal-ceramic and all-ceramic FPDs over a defined follow-up period. The objectives were to assess patient satisfaction with esthetics, function, comfort, and overall QoL using validated instruments, identify any differences in perceived outcomes between the two restorative materials, and correlate these subjective measures with objective clinical parameters, such as survival and complications.

## Materials and methods

Study design

This prospective observational study was conducted in the Department of Prosthodontics, RKDF Dental College and Research Centre, Bhopal, India, from March 2022 to December 2023, to compare PROMs between metal-ceramic and all-ceramic FPDs over a 12-month follow-up period. The choice of restorative material (metal-ceramic or all-ceramic) was determined by standard clinical decision-making, including patient preferences, esthetic demands, occlusion, economic considerations, and contraindications, without investigator-driven randomization or allocation. This observational approach allowed for the evaluation of real-world clinical practice outcomes. Ethical approval was obtained from the Institutional Review Board, and all participants provided written informed consent prior to enrolment. Follow-up assessments were scheduled at baseline (immediately post-insertion), 6, and 12 months to capture longitudinal changes in the PROMs.

Participants and sample size

A priori sample size calculation was performed using G*Power software (version 3.1.9.7; Heinrich Heine University Düsseldorf, Düsseldorf, Germany). To detect a medium effect size (d=0.6), derived from a relevant reference study, with a statistical power of 80% and an alpha error set at 5%, a minimum total sample size of 72 participants was determined [7]. Anticipating a potential 20% attrition rate, the target enrolment was increased to 86 participants to ensure adequate power at the end of the study. The participants were equally allocated to two study groups: metal-ceramic crowns and all-ceramic crowns.

Inclusion and exclusion criteria

Participants were adults aged 18-60 years with good oral hygiene, adequate bone support for abutment teeth, and no contraindications to fixed prosthodontics. The inclusion criteria were the need for a three- or four-unit FPD in the premolar-molar area, vital or endodontically treated abutment teeth without periapical pathology, and opposing natural dentition or fixed restorations. To minimize variability related to opposing dentition, only patients with opposing natural teeth or fixed restorations were included, while those with removable prostheses or severely worn occlusal surfaces were excluded. All restorations were placed in the posterior premolar-molar region, and occlusion was standardized at insertion by establishing even centric contacts and eliminating eccentric interferences using a uniform clinical protocol. This approach ensured functional comparability of antagonists while reflecting routine clinical conditions. The exclusion criteria encompassed patients with severe bruxism, periodontal disease (probing depth >4 mm), allergies to restorative materials, pregnancy, or systemic conditions affecting oral health, such as uncontrolled diabetes.

Restorative materials and procedures

For the metal-ceramic group (n = 43), FPDs were fabricated using a high-noble alloy framework (IPS d.SIGN 91; Ivoclar Vivadent AG, Schaan, Liechtenstein) veneered with feldspathic porcelain (IPS Inline; Ivoclar Vivadent AG, Schaan, Liechtenstein). The alloy was cast using the lost-wax technique, and porcelain was layered and fired according to the manufacturer’s protocols to achieve optimal esthetics and strength. In the all-ceramic group (n = 43), restorations were constructed using yttria-stabilized zirconia frameworks (Lava Plus Zirconia; 3M ESPE, St. Paul, MN, USA) milled via computer-aided design/computer-aided manufacturing (CAD/CAM) using a dental milling unit (inLab MC X5; Dentsply Sirona, Charlotte, NC, USA), followed by veneering with compatible porcelain (VITA VM 9; VITA Zahnfabrik, Bad Säckingen, Germany). Impressions were obtained using polyvinyl siloxane material (Aquasil Ultra, Dentsply Sirona, Charlotte, NC, USA), and provisional restorations were provided during the fabrication period. Cementation was performed using dual-cure resin cement (RelyX Ultimate, 3M ESPE, St. Paul, MN, USA) under rubber dam isolation, ensuring standardized occlusal adjustments and polishing for both groups.

Outcome measures and data collection

Patient-reported outcome measures were assessed using validated instruments, including the Oral Health Impact Profile-14 (OHIP-14) questionnaire [8], which evaluates seven domains of oral health-related QoL on a Likert scale ranging from 0 (never) to 4 (very often), with lower scores indicating better outcomes. Additionally, visual analog scales (VAS) were used for patient satisfaction ratings in esthetics, function, comfort, and overall perception, ranging from 0 (completely dissatisfied) to 10 (completely satisfied) [9]. Permissions to use the OHIP-14 questionnaire and VAS were obtained. Clinical examinations recorded objective parameters, such as survival rates, chipping, fractures, and secondary caries. Data were collected at each follow-up visit by blinded evaluators for clinical assessments to minimize bias. All tools were administered in the local language after cultural adaptation and validation of the questionnaires.

Statistical analysis

Data were then analyzed using statistical software (IBM SPSS Statistics version 23 (IBM Corp., Armonk, USA)). The normality of the distributions of the OHIP and VAS scores was assessed using the Shapiro-Wilk test, which indicated non-normal data. Consequently, non-parametric tests were employed: intergroup comparisons were made using the Mann-Whitney U test, and intragroup comparisons across multiple time points were analyzed using the Friedman test. Categorical variables were evaluated using the chi-square test. Statistical significance was set at p < 0.05.

## Results

Six patients (three per group) were lost to follow-up; therefore, the final study was conducted on 80 patients (40 patients per group). The study comprised 86 patients, of whom 80 (93%) completed follow-up, indicating a low attrition rate. Participants had a mean age of 35.3±11.2 years, with a slightly higher proportion of males than females. The primary indication for crown placement was root canal treatment, followed by tooth fractures and developmental defects. This demographic and clinical profile suggests that the sample represents a typical adult patient population requiring single-tooth restorations, with outcomes primarily applicable to cases involving endodontically treated teeth (Table 1).

**Table 1:** Baseline demographic and clinical characteristics of the study population. Values are expressed as mean ± standard deviation (SD) or frequency (percentage).

Characteristic	Value
Total patients	86 (100%)
Patients completed follow up	80 (93%)
Male/Female	44 (55%)/36 (45%)
Mean age (Years)	35.3±11.2
Reasons for crown placement	
Fracture of tooth crown	18 (23%)
Root canal treatment	54 (67%)
Developmental defect in tooth crown	8 (10%)

The demographic variable of sex was similarly distributed between the groups, showing no significant association (p=0.654). A significant difference was observed in the incidence of chipping, which was higher in the all-ceramic group than in the metal-ceramic group (p=0.026). The fracture rates were comparable between the groups (p=0.363). Although not statistically significant at the 0.05 threshold, a trend toward a higher occurrence of secondary caries was noted in the metal-ceramic group (Table 2).

**Table 2:** Distribution of categorical clinical outcomes between study groups. Categorical variables compared using the Chi-square test. *Statistically significant at p < 0.05.

Variables	Category	Metal-Ceramic, n=40	All-Ceramic, n=40	Chi stat	df	P value
		n (%)	n (%)			
Sex	Male	25 (62.5)	19 (47.5)	0.41	1	0.654
	Female	15 (37.5)	21 (52.5)			
Chipping	Yes	7 (17.5)	16 (40.0)	4.94	1	0.026*
	No	33 (82.5)	24 (60.0)			
Fracture	Yes	8 (20.0)	5 (12.5)	0.83	1	0.363
	No	32 (80.0)	35 (87.5)			
Secondary caries	Yes	13 (26.0)	6 (15.0)	3.38	1	0.066
	No	27 (74.0)	34 (85.0)			

At baseline, there were no statistically significant differences between the groups for either the OHIP-14 total score (p=0.353) or the VAS satisfaction score (p=0.676), indicating comparable initial oral health-related quality of life (QoL) and patient satisfaction. However, significant intergroup differences were observed at the 6- and 12-month follow-ups. The all-ceramic group demonstrated significantly lower (better) OHIP-14 median scores at 6 and 12 months, reflecting superior long-term oral health impact. Similarly, the VAS satisfaction scores were significantly higher (better) for the all-ceramic group at both 6 and 12 months. These results suggest that restorations with all-ceramic materials were associated with significantly better PROMs in terms of both QoL and satisfaction over a one-year period compared to metal-ceramic restorations (Table 3).

**Table 3:** Intergroup comparison of OHIP-14 and VAS scores using the Mann–Whitney U test. Non-parametric intergroup comparisons performed using the Mann–Whitney U test. *p < 0.05 was considered statistically significant. Oral Health Impact Profile-14 (OHIP-14) questionnaire [8] and visual analog scale (VAS) [9] were used for assessment.

Outcome	Group	Median	Interquartile range (IQR)	U	p-value
OHIP-14 (Baseline)	Metal-Ceramic	26	24-32	704	0.353
	All-Ceramic	26	23-29		
OHIP-14 (6 months)	Metal-Ceramic	8	6.7-12.0	367	0.001*
	All-Ceramic	6	5-7		
OHIP-14 (12 months)	Metal-Ceramic	15	12-18	187.5	0.001*
	All-Ceramic	8	7-9		
VAS score (Baseline)	Metal-Ceramic	8	7-8	758.5	0.676
	All-Ceramic	8	7-8		
VAS score (6 months)	Metal-Ceramic	4	4-5	309	0.001*
	All-Ceramic	3	2-4		
VAS score (12 months)	Metal-Ceramic	4	4-5	430.5	0.001*
	All-Ceramic	3	3-4		

**Figure 1:**
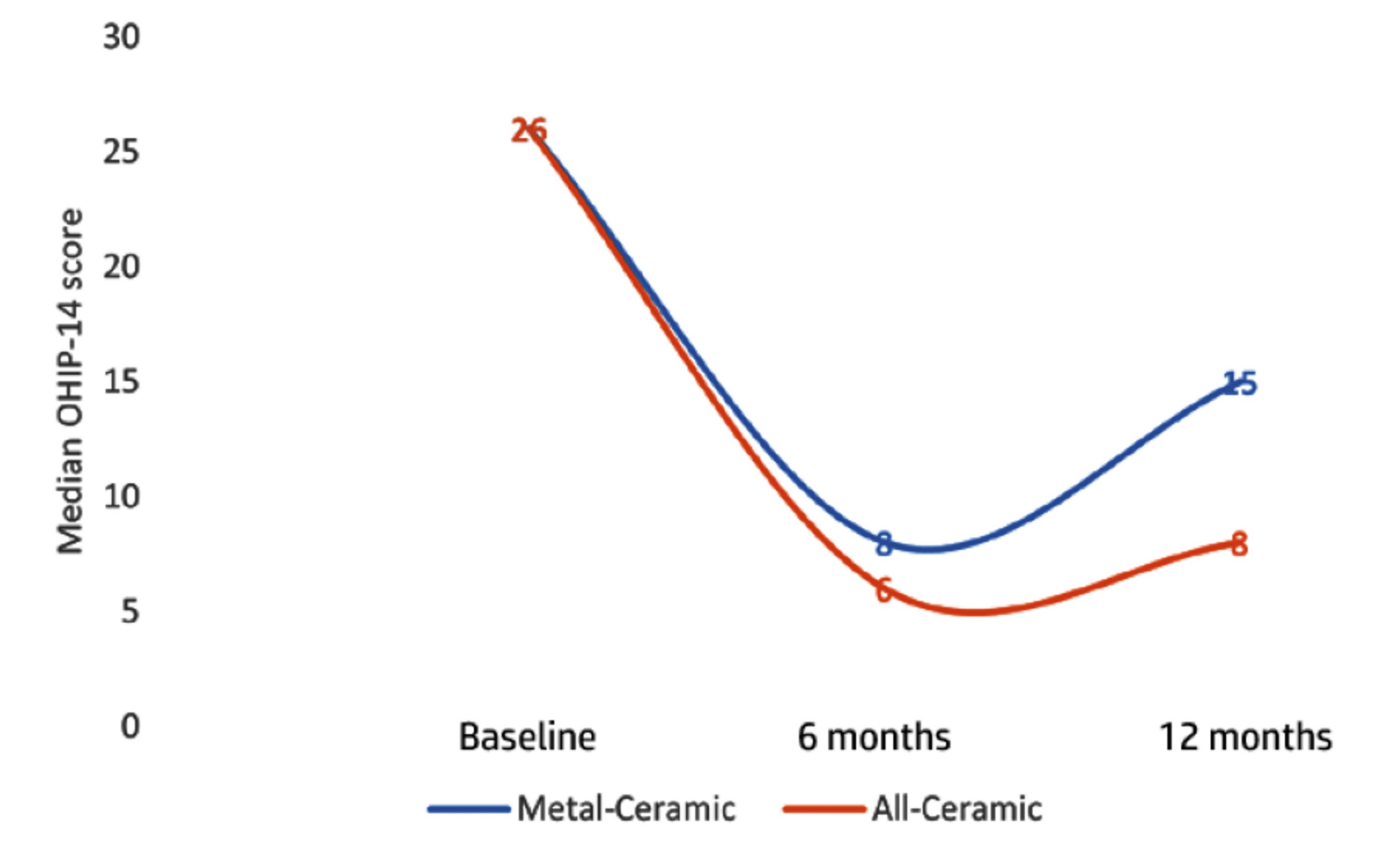
Line graph showing the median Oral Health Impact Profile-14 (OHIP-14) score at different time intervals.

The Friedman test revealed a statistically significant change in the OHIP-14 scores over time within both the metal-ceramic (p<.001) and all-ceramic (p<.001) groups. For the metal-ceramic group, scores improved from a median of 26 at baseline to 8 at 6 months but worsened to 15 by 12 months. In contrast, the all-ceramic group showed sustained improvement, with medians decreasing from 26 to 6 and then to eight at the same intervals (Figure 1).

This suggests that while both materials provided initial QoL benefits, all-ceramic restorations were associated with more favorable and stable long-term patient-reported outcomes (Table 4).

**Table 4:** Intragroup comparison of OHIP-14 scores across time using the Friedman test. Dunn’s post hoc test was applied following the Friedman test. *p < 0.05 indicates statistically significant pairwise difference; Oral Health Impact Profile-14 (OHIP-14) questionnaire [8] was used.

Time Points	Metal-Ceramic					All-Ceramic				
	Median	IQR	Friedman χ²	df	P value	Median	IQR	Friedman χ²	df	P value
OHIP-14 total score (Baseline)	26	(24-32)	63.71	2	0.001*	26	(23-29)	65	2	0.001*
OHIP-14 total score (6 months)	8	(6.75-12)				6	(5-7)			
OHIP-14 total score (12 months)	15	(12-18)				8	(7-9)			

The post hoc Dunn test revealed that for both groups, the OHIP-14 scores showed a highly significant improvement (p<0.001) from baseline to both the 6-month and 12-month follow-ups. However, a key divergence was observed in the trajectories between the later intervals. A statistically significant worsening in scores occurred from 6 to 12 months in the metal-ceramic group (p=0.004). In contrast, this decline was not statistically significant in the all-ceramic group (p=0.076). This suggests that all-ceramic restorations provided a more stable and sustained improvement in patient-reported oral health over one year (Table 5).

**Table 5:** Post hoc Dunn test for pairwise comparison of OHIP-14 scores. Dunn’s post hoc test was applied following the Friedman test. *p < 0.05 indicates statistically significant pairwise difference; Oral Health Impact Profile-14 (OHIP-14) questionnaire [8] was used.

Comparison	Metal-Ceramic		All-Ceramic	
	Z stats	P value	Z stats	P value
Baseline vs. 6 months	1.76	0.001*	1.75	0.001*
Baseline vs. 12 months	1.13	0.001*	1.25	0.001*
6 months vs. 12 months	-0.64	0.004*	-0.50	0.076

**Figure 2:**
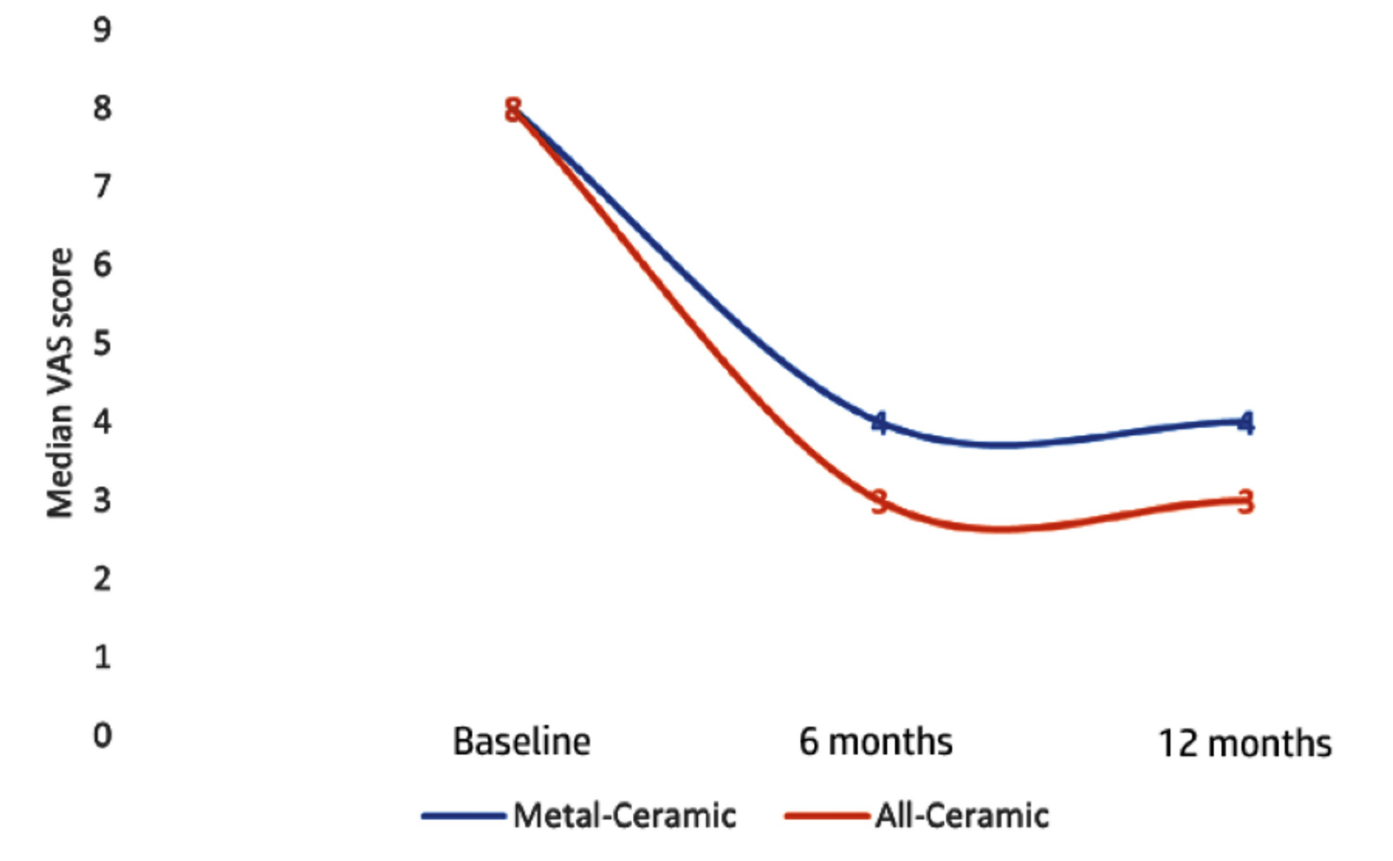
Line graph showing the median visual analog scale (VAS) score at different time intervals.

The Friedman test confirmed a statistically significant change in patient satisfaction (VAS scores) over time in both groups. While both groups started with identical median scores of 8, their trajectories diverged during follow-up. Satisfaction declined in the metal-ceramic group to a median of 4 at 6 and 12 months. In contrast, the all-ceramic group achieved and maintained a lower (better) median score of 3 at the same time intervals (Figure 2).

This indicates that all-ceramic restorations were associated with significantly higher and more stable long-term patient satisfaction than metal-ceramic alternatives (Table 6).

**Table 6:** Intragroup comparison of VAS scores across time using Friedman test. The Friedman test was used for repeated measures within each group. *Statistically significant change over time (p < 0.05); the visual analog scale (VAS) [9] was used for assessment.

Time point	Metal-Ceramic					All-Ceramic				
	Median	IQR	Friedman χ²	df	P value	Median	IQR	Friedman χ²	df	P value
VAS score (Baseline)	8	(7-8)	58.76	2	0.001*	8	(7-8)	61.91	2	0.001*
VAS score (6 months)	4	(4-5)				3	(2-4)			
VAS score (12 months)	4	(4-5)				3	(3-4)			

Post hoc analysis with the Dunn test revealed that patient satisfaction (VAS scores) significantly decreased from baseline to both the 6-month and 12-month follow-ups for both restoration groups (all p<.001). The key finding was the stability of satisfaction scores between later time points. No significant change was observed from 6 to 12 months for the metal-ceramic group (p=1.000), and the change in the all-ceramic group was also non-significant (p=0.195). This suggests that the initial adjustment period significantly influenced satisfaction, after which scores stabilize, with all-ceramic restorations maintaining a superior satisfaction level throughout the follow-up period (Table 7).

**Table 7:** Post hoc Dunn test for pairwise comparison of VAS scores. Dunn’s post hoc test was used for multiple comparisons. *p < 0.05 indicates statistical significance; the visual analog scale (VAS) [9] was used for assessment.

Pairwise Comparison	Metal-Ceramic		All-Ceramic	
	Z stats	P value	Z stats	P value
Baseline vs. 6 months	1.54	0.001*	1.69	0.001*
Baseline vs. 12 months	1.42	0.001*	1.27	0.001*
6 months vs. 12 months	-0.11	1	-0.41	0.195

## Discussion

This prospective clinical study evaluated and compared the PROMs of metal-ceramic and all-ceramic FPDs over a one-year follow-up period. The findings demonstrated that while both restorative materials significantly improved oral health-related quality of life and patient satisfaction compared to the baseline, all-ceramic FPDs were associated with superior and more stable long-term PROMs, particularly at 6 and 12 months.

At baseline, no statistically significant differences were observed between the two groups in the OHIP-14 or VAS scores, indicating comparable pre-treatment expectations and initial oral health perceptions. This similarity strengthens the internal validity of the study by suggesting that post-treatment differences were attributable to the restorative material rather than to baseline bias. The significant improvement in PROMs in both groups at 6 months reflects the well-documented positive impact of fixed prosthodontic rehabilitation on function, comfort, and psychosocial well-being, irrespective of the material choice [7,8,10].

However, a divergence in patient-reported outcomes became evident at later follow-ups. The all-ceramic group demonstrated significantly lower OHIP-14 scores and better VAS satisfaction at both 6 and 12 months, whereas the metal-ceramic group showed a partial deterioration in QoL scores between 6 and 12 months. The differences observed between groups align with the domains assessed by the OHIP-14 and VAS instruments. The sustained improvement in the all-ceramic group may be attributed to favorable perceptions of esthetics, comfort, and social confidence, influencing the psychological discomfort, social disability, and handicap domains of OHIP-14. In contrast, the partial deterioration in QoL scores in the metal-ceramic group between 6 and 12 months may reflect increased patient awareness of esthetic limitations or minor functional perceptions over time, despite acceptable clinical performance. The VAS findings corroborate this trend, as esthetics and comfort, key patient-centered variables, remained higher and more stable in the all-ceramic group. This pattern suggests that although metal-ceramic restorations provide effective short-term rehabilitation, their long-term patient-perceived performance may be less favorable [11,12].

The superior PROMs observed with all-ceramic restorations can be attributed to several factors. First, all-ceramic systems offer enhanced esthetics because of their translucency and absence of metal substructures, which contribute to improved patient confidence and social comfort [5]. Second, improved biocompatibility and reduced risk of gingival discoloration may enhance long-term comfort and periodontal perceptions. Third, advancements in zirconia-based materials and CAD/CAM (Computer-Aided Design / Computer-Aided Manufacturing) fabrication have improved the marginal accuracy and occlusal precision, which may positively influence functional satisfaction [13].

Interestingly, the clinical complication profile also provided insights into the PROMs findings. Metal-ceramic restorations have traditionally demonstrated high survival rates, and the present study identified a significantly higher incidence of chipping in the all-ceramic group. This finding was supported by previous studies, where lower risks of complications and chipping were noted for metal-ceramic restorations compared to all-ceramic restorations [2,14,15]. All-ceramic fixed partial dentures, particularly veneered zirconia systems, have a high elastic modulus and rigid core, which transfers occlusal stresses to the veneering ceramic. The veneering porcelain is more brittle and has lower fracture toughness, making it more susceptible to cohesive chipping, especially under posterior occlusal loads. In contrast, metal-ceramic restorations benefit from a ductile metal substructure, which absorbs and distributes functional stresses more evenly, reducing stress concentration within the veneering porcelain. Additionally, mismatches in coefficients of thermal expansion, residual firing stresses, and veneer thickness variations are more critical in all-ceramic systems. These factors, combined with parafunctional micro-loading over time, explain why all-ceramic restorations may show higher chipping rates despite comparable survival and patient-reported outcomes.

Intragroup analysis further reinforced these observations. Both groups showed significant improvements in the OHIP-14 and VAS scores from baseline to follow-up. However, the metal-ceramic group exhibited a statistically significant decline in oral health-related QoL between 6 and 12 months, whereas the all-ceramic group maintained stable outcomes. This sustained improvement highlights the potential of all-ceramic restorations to provide consistent patient satisfaction over time, aligning with contemporary expectations for esthetic and functional dental care [5].

Despite the significantly higher incidence of chipping observed in the all-ceramic group compared with the metal-ceramic group, the clinical relevance of this finding should be interpreted with caution. Veneer chipping in all-ceramic FPDs is most often a minor technical complication that is frequently amenable to chairside polishing or limited repair, without necessitating prosthesis replacement or compromising function. Importantly, in the present study, the higher chipping rate did not adversely affect patient-reported outcomes; on the contrary, all-ceramic restorations demonstrated superior and more stable QoL and satisfaction scores. This suggests that patients may place greater value on esthetic integration, translucency, and metal-free restorations than on minor technical complications that do not impair comfort or function. Clinically, these findings highlight a well-recognized trade-off between enhanced esthetics and a modest increase in technical complications, reinforcing the importance of shared decision-making in material selection, particularly for patients with high esthetic expectations.

Clinical implications

From a clinical perspective, these findings have important implications for the selection of materials in fixed prosthodontics. While metal-ceramic FPDs remain a reliable and cost-effective option with proven longevity, clinicians should recognize that all-ceramic restorations may offer superior patient-perceived outcomes, particularly for patients with high esthetic demands or sensitivity to long-term comfort and appearance. Incorporating PROMs into routine clinical evaluations can facilitate shared decision-making, enabling clinicians to align treatment choices with patient expectations and preferences, rather than relying solely on technical performance indicators.

Limitations

This study has several limitations. First, the observational design without randomization introduces potential selection bias, as material choice was influenced by clinical judgment and patient preferences. Second, the follow-up period was limited to one year; longer observation is necessary to assess whether PROMs differences persist over extended periods and how they relate to long-term survival. Third, PROMs, while invaluable, are inherently subjective and may be influenced by psychological, social, and cultural factors that were not fully controlled in this study. Finally, this study focused on posterior FPDs, and the findings may not be directly generalizable to anterior restorations with higher esthetic demands.

In addition to the limitations already noted, the study did not account for demographic and behavioral variables such as age, sex, dietary habits, or parafunctional activity, which may influence patient perception and PROM responses. The absence of domain-wise or multivariate analysis limits deeper interpretation of how individual OHIP-14 domains contributed to overall scores. Furthermore, as PROMs are inherently subjective, inter-individual variability in perception may affect reproducibility despite the use of validated questionnaires. These factors should be addressed in future studies through stratified analyses and longer follow-up.

Future directions

Future randomized controlled trials with longer follow-up periods and the integration of both PROMs and objective clinical outcomes are warranted. The inclusion of cost-effectiveness analyses and qualitative patient interviews may further enrich the understanding of patient-centered success in prosthodontic rehabilitation. Despite extensive literature comparing conventional metal-ceramic and all-ceramic fixed partial dentures, evidence remains limited regarding patient-reported outcomes and technical complications associated with porcelain-veneered frameworks fabricated using Direct Metal Laser Sintering (DMLS) technology. Moreover, the influence of dietary habits, including consumption of hard, fibrous, or culturally specific foods, on veneer chipping, functional comfort, and long-term satisfaction has not been adequately explored. This gap is particularly relevant in populations with diverse food textures and chewing patterns, where occlusal loading characteristics may differ significantly. Therefore, further clinical studies incorporating PROMs, material-specific fabrication techniques such as DMLS, and contextual factors like food habits are needed to better inform material selection and patient-centered prosthodontic care.

## Conclusions

Within the limitations of this prospective clinical study, both metal-ceramic and all-ceramic fixed partial dentures significantly improved oral health-related QoL and patient satisfaction compared to baseline. However, all-ceramic restorations demonstrated superior and more stable patient-reported outcomes over a one-year follow-up period, particularly with respect to comfort, esthetics, and overall satisfaction. Although metal-ceramic restorations remain a reliable treatment option with acceptable clinical performance, these findings emphasize the growing importance of patient-centered outcome measures in prosthodontic decision-making. Therefore, all-ceramic FPDs may be considered a preferable option when long-term patient perception and quality of life are prioritized.
